# Using fMRI to characterize how cortex represents limb motions

**DOI:** 10.1186/1471-2202-15-S1-P126

**Published:** 2014-07-21

**Authors:** Samir Menon, Jack Zhu, Paul Quigley, Franco Pestilli, Kwabena Boahen, Oussama Khatib

**Affiliations:** 1 Department of Computer Science, Stanford University, Stanford, CA, 94305, USA; 2 Department of Psychology, Stanford University, Stanford, CA, 94305, USA; 3 Department of Bioengineering, Stanford University, Stanford, CA, 94305, USA

## 

Neuroimaging experiments that map limb motions on to the brain observe fractured somatotopic maps, with correlated neural responses across functionally related joints in the arm [1]. Analyzing such experiments involves visually comparing winner-takes-all neural activation maps for different subjects that are generated with generalized linear models [2]. Such analyses, however, abstract cross-joint correlations and treat reliable deviations from canonical neural (haemodynamic) response functions as temporal noise. Here, using classification accuracy while delineating different limb motions as a metric, we demonstrate that the peak neural response amplitude---upon which winner-takes-all analyses are based---is the least informative part of the time-series. In contrast, our experiments suggest that neural responses are most informative after the initial response peak (t=4-10s). Our observations extend to primary motor (M1), pre-motor (PMd), somatosensory (S1), superior parietal (SupPar), and supplementary motor (SMA) cortices, matching prior region-agnostic results [3]. As expected for open-loop limb motions, median M1 and S1 classification accuracies are greater than SupPar, PMd and SMA. All accuracies exceed the ventricles, which set a data-driven noise threshold at chance (50-55% accuracy; chance=50%) and demonstrate that our datasets lack task-correlated noise.

Our results suggest that Functional Magnetic Resonance Imaging (fMRI) time-series responses convey sufficient information to classify a variety of motor tasks in regions where neural activity is expected to be correlated across conditions. Reproducing our results, however, may require fMRI datasets with minimal (<1mm) head-motion, no spatial smoothing, and tests for null (baseline) results in regions with no expected effect.

## Methods

We used fMRI to scan three subjects who moved their wrist, elbow or shoulder up and down, or rotated their wrist or shoulder while holding two different weights (50g, 500g. 8s*32trials*10conditions). We used Freesurfer's Desikan--Killiany atlas to identify motor regions and iterated over each region in 1cm^3^ sections, using randomly sampled voxels (40 bootstraps; 50 of ~150 voxels) and a maximum-margin hyperplane with leave-one-out cross-validation to pair-wise classify limb motions. We also compared random (unbiased) responses with reliable voxels selected using a traditional general linear impulse response model.

**Figure 1:**
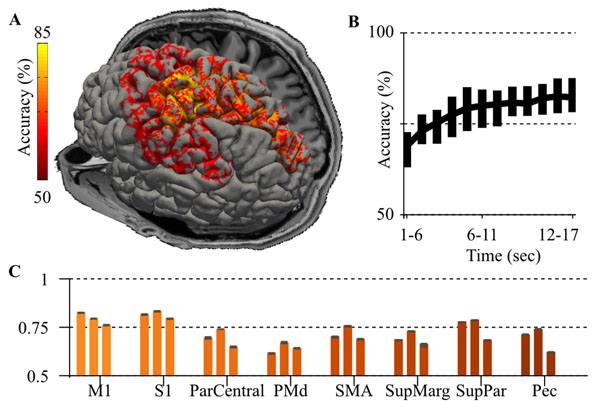
**Motor classification. A.** Classification accuracy by region for a subject. **B.** Later stage fMRU neural responses improve classification accuracy. A sliding window of time-series data used increases accuracy till 15 seconds. **C.** Medican and 95%ile accuracies are shown for three subjects across a variety of regions.
